# Mercury-Supported Biomimetic Membranes for the Investigation of Antimicrobial Peptides

**DOI:** 10.3390/ph7020136

**Published:** 2014-01-23

**Authors:** Lucia Becucci, Rolando Guidelli

**Affiliations:** 1 Department of Chemistry “Ugo Schiff”, Florence University, Via della Lastruccia 3, Sesto Fiorentino (Firenze) 50019, Italy; 2 Retired professor from Florence University, Firenze 50121, Italy; E-Mail: rolando.guidelli@libero.it

**Keywords:** tethered bilayer lipid membranes, self-assembled monolayers, electrochemical impedance spectroscopy, potential-step chronocoulometry, cyclic voltammetry

## Abstract

Tethered bilayer lipid membranes (tBLMs) consist of a lipid bilayer interposed between an aqueous solution and a hydrophilic “spacer” anchored to a gold or mercury electrode. There is great potential for application of these biomimetic membranes for the elucidation of structure-function relationships of membrane peptides and proteins. A drawback in the use of mercury-supported tBLMs with respect to gold-supported ones is represented by the difficulty in applying surface sensitive, spectroscopic and scanning probe microscopic techniques to gather information on the architecture of these biomimetic membranes. Nonetheless, mercury-supported tBLMs are definitely superior to gold-supported biomimetic membranes for the investigation of the function of membrane peptides and proteins, thanks to a fluidity and lipid lateral mobility comparable with those of bilayer lipid membranes interposed between two aqueous phases (BLMs), but with a much higher robustness and resistance to electric fields. The different features of mercury-supported tBLMs reconstituted with functionally active membrane proteins and peptides of bacteriological or pharmacological interest may be disclosed by a judicious choice of the most appropriate electrochemical techniques. We will describe the way in which electrochemical impedance spectroscopy, potential-step chronocoulometry, cyclic voltammetry and phase-sensitive AC voltammetry are conveniently employed to investigate the structure of mercury-supported tBLMs and the mode of interaction of antimicrobial peptides reconstituted into them.

## 1. Introduction

In view of the complexity and diversity of the functions performed by the different peptides and proteins embedded in a biomembrane it has been found expedient to incorporate them singly into experimental models of cell membranes, so as to isolate and investigate their functions. This serves to reduce complex membrane processes to well-defined interactions between selected biomolecules, lipids and ligands. There is great potential for application of experimental models of biomembranes (the so-called biomimetic membranes) for the elucidation of structure-function relationships of many biologically important membrane peptides and proteins. With only a few exceptions, biomimetic membranes consist of a more or less complex architecture that includes a lipid bilayer.

**Figure 1:**
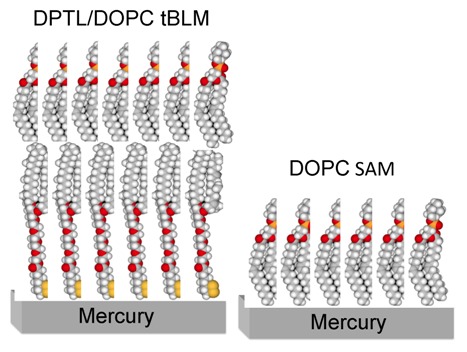
Schematic picture of a phosphatidylcholine (DOPC) self-assembled monolayer (**right**) and of a DPTL/DOPC tethered bilayer lipid membrane (**left**). Hydrogen atoms are marked in white, carbon atoms in gray, oxygen atoms in red, phosphorus atoms in orange and sulfur atoms in yellow.

Tethered bilayer lipid membranes (tBLMs) are versatile model systems that provide a defined platform for the incorporation of peptides and proteins and for the investigation of their functional activity [1,2,3]. They are obtained by tethering a “thiolipid” monolayer to the surface of a noble metal such as Au, Ag or Hg. Thiolipid molecules consist of a hydrophilic chain (the spacer) terminated at one end with a sulfhydryl or disulfide group for anchoring to the support and covalently linked at the other end to two alkyl chains simulating the hydrocarbon tails of a lipid. A tethered thiolipid monolayer exposes a hydrophobic surface to the bulk aqueous phase and provides one half of the lipid bilayer. The other half is obtained by forming a lipid monolayer on top of the thiolipid monolayer. A particularly convenient thiolipid, 2,3-di-*O*-phytanyl-sn-glycerol-1-tetraethylene-glycol-d,l-α lipoic acid ester lipid, called DPTL [4], consists of a tetraethyleneoxy (TEO) hydrophilic chain, terminated at one end with a lipoic acid residue for anchoring to the metal surface, and covalently linked at the other end to two phytanyl chains. Figure 1 shows a schematic picture of a tBLM consisting of a DPTL thiolipid monolayer anchored to mercury, with a dioleoylphosphatidylcholine (DOPC) monolayer on top of it.

With respect to solid metal supports such as gold or silver, mercury has the advantage of providing a defect free, fluid and readily renewable surface to the self-assembling thiolipid/lipid bilayer. Moreover, it imparts to the lipid molecules of the whole bilayer a lateral mobility comparable with that of biomembranes. The free movement of lipid molecules enables mercury-supported tBLMs to react to the presence of proteins, charges and physical forces in a dynamic and responsive manner. A satisfactory fluidity allows these biomimetic membranes to reorganize upon interaction with external perturbations, mimicking the functionality of living cell membranes. In particular, lateral mobility enables Hg-supported tBLMs to incorporate membrane proteins, such as the Human Ether-à-go-go-Related Gene (HERG) potassium channel [5] from their detergent solutions by making space for them; it also determines the spontaneous separation of the components of a lipid mixture (demixing), giving rise to the formation of lipid microdomains called lipid rafts [6].

Mercury-supported DPTL/phospholipid tBLMs are readily prepared by immersing a hanging mercury drop electrode (HMDE) into an ethanol solution of DPTL for about 20 min to anchor a thiolipid monolayer on the mercury surface. A phospholipid monolayer is then self-assembled on top of the thiolipid monolayer by simply immersing the thiolipid-coated mercury drop in an aqueous electrolyte on whose surface a lipid film has been previously spread [7]. Thanks to the hydrophobic interactions between the alkyl chains of the thiolipid and those of the lipid, this simple procedure gives rise to a lipid bilayer anchored to the mercury surface via the hydrophilic spacer moiety of the thiolipid, which is in contact with the mercury surface; the spacer is the place where ions can be attracted and accommodated under the influence of an electric field. All mercury-supported tBLMs to be described in the following part of this review consist of a DPTL anchored to the surface of a HMDE, with a DOPC or diphytanoylphosphatidylcholine (DPhyPC) monolayer self-assembled on top of it, unless otherwise stated. The electric potential *E* applied to this tBLM can be related to the corresponding transmembrane potential *ϕ*_trans_, namely the potential difference across the sole lipid bilayer moiety of the tBLM. In the absence of ion channels in the lipid bilayer moiety, and hence in the absence of ions in the hydrophilic spacer, *ϕ*_trans_ can be estimated from the equation *ϕ*_trans_ = 0.72 × (*E* (*vs.* Ag|AgCl|0.1M KCl) + 0.450 V), derived on the basis of an approximate extra-thermodynamic procedure [8]. Incidentally, a possible procedure to estimate *ϕ*_trans_ at a DPTL tethered to a Au(111) electrode from the potential of zero free charge measured by the immersion method was recently proposed by Lipkowski and coworkers [9]. In what follows, all applied potentials are referred to the Ag|AgCl|0.1M KCl reference electrode.

A more easily prepared but less versatile mercury-supported biomimetic membrane consists of a phospholipid monolayer directly self-assembled on a HMDE [10]. The lipid coating is obtained by spreading a solution of the lipid in pentane on the surface of an aqueous electrolyte, allowing the pentane to evaporate and immersing a HMDE in the electrolyte. This procedure gives rise to a lipid self-assembled monolayer (SAM), with the hydrocarbon tails directed toward the hydrophobic mercury surface and the polar heads directed toward the solution. A schematic picture of a mercury-supported DOPC SAM is shown in Figure 1. The defect-free support provided by liquid mercury to the lipid monolayer and the complete absence of solvent in the film impart high fluidity, lipid lateral mobility, mechanical stability, resistance to electric fields and reproducibility to the monolayer. This self-assembly procedure exploits the fact that mercury is the most hydrophobic metal. Even though this biomimetic membrane mimics only one half of a cell membrane and has no ionic reservoir on the metal side of the lipid monolayer, under certain conditions it can be used for investigating the properties of channel-forming peptides such as gramicidin [11,12] and polyene antifungal drugs such as amphotericin B [13,14], which have a length comparable with the monolayer thickness (~3.2 nm). The ability of short peptides to form pores or channels in the membrane can be tested by verifying whether they allow the electroreduction of inorganic cations, such as Tl^+^ [11,12,15] and Cd^2+^ [14,16], whose flux toward the mercury surface is blocked by the SAM over the whole potential range of stability of the latter. In this case, the whole mercury drop provides a large ionic reservoir, where desolvated metal ions are accommodated in a sea of free electrons, with amalgam formation. The effect of longer peptides [17] or small proteins [18] on a lipid monolayer may also provide useful information on their initial interaction with the outer leaflet of a biological membrane.

A drawback with the use of mercury-supported tBLMs with respect to gold-supported ones is represented by the notable difficulty in applying surface sensitive, spectroscopic and scanning probe microscopic techniques to obtain exhaustive information on the architecture of these biomimetic membranes. Thus, e.g., scanning tunneling microscopy (STM) allows a direct visualization of pores of antibiotic peptides such as alamethicin [19] and gramicidin [20] in gold-supported planar phospholipid matrixes, surface plasmon resonance (SPR) measures the optical thickness of tBLMs self-assembled on gold and silver [21], neutron reflectivity (NR) estimates the profile of water content along the spacer and the lipid bilayer moiety of gold-supported tBLMs [22], and polarization modulation Fourier transform infrared reflection adsorption spectroscopy (PM-FTIRRAS) is used to investigate the electric field driven transformations of gold-supported phospholipid bilayers [23]. Nonetheless, mercury-supported tBLMs are definitely superior to gold-supported biomimetic membranes for the investigation of the function of membrane peptides and proteins, thanks to a fluidity and lipid lateral mobility comparable with those of bilayer lipid membranes interposed between two aqueous phases (BLMs), but with a much higher robustness and resistance to electric fields. The different features of Hg-supported biomimetic membranes reconstituted with functionally active membrane proteins and peptides of bacteriological or pharmacological interest may be disclosed by a judicious choice of the most appropriate electrochemical techniques. Of the numerous electrochemical techniques, we will consider four that are particularly relevant to this scope, *i.e.*, electrochemical impedance spectroscopy (EIS), potential-step chronocoulometry, phase-sensitive AC voltammetry, and the most familiar technique among researchers with a rudimentary background in freshman electrochemistry, namely cyclic voltammetry (CV). In this contribution we will describe the way in which these different techniques are conveniently employed to investigate the structure of mercury-supported tBLMs and the mode of interaction of antimicrobial peptides reconstituted into them.

## 2. Electrochemical Impedance Spectroscopy

Electrochemical impedance spectroscopy (EIS) is the technique of choice for discriminating the various sections of a tBLM on the basis of their different dielectric properties and for monitoring the way in which these properties are affected by the incorporation and functional activity of membrane peptides and proteins. EIS consists in perturbing the system under investigation (the biomimetic membrane) with an electric potential generated by superimposing to a constant applied potential *E* an AC voltage of small amplitude (~10 mV peak to peak), whose frequency, *f*, is progressively varied from 10^5^ to 10^−3^ Hz. For each frequency, the EIS instrument measures the amplitude, *I*, and the phase shift *φ*, of the current flowing through the electrochemical cell with the same frequency, thus generating an impedance spectrum. The current may be displayed by reporting its “in-phase” component, *I* cos *φ*, along the abscissa of an orthogonal coordinate system (the “complex plane”) and its “quadrature” component, *I* sin *φ*, along the ordinate.

Application of an AC voltage of amplitude *V* and frequency *f* to a pure resistor of resistance *R* yields a current of equal frequency *f* and of amplitude *V/R*, in phase with the voltage. Conversely, application of the AC voltage to a pure capacitor of capacitance *C* yields a current of frequency *f* and amplitude 2π*fC*, out of phase by −π/2 with respect to the voltage, *i.e.*, “in quadrature” with it. This state of affairs can be expressed by stating that the admittance *Y* of a resistive element equals 1/*R*, whereas that of a capacitive element equals −i*ωC*, where *ω* = 2π*f* is the angular frequency. More generally, in an “equivalent circuit” consisting of resistances and capacitances, *Y* is a complex quantity, and the impedance *Z* is equal to 1/*Y*, by definition. Hence, *Z* equals *R* for a resistive element, and i/*ωC* for a capacitive element. In analogy with the resistance in direct current measurements, the overall impedance of two circuit elements in series is equal to the sum of the impedances of the single circuit elements. Conversely, the overall impedance of two circuit elements in parallel is such that its reciprocal is equal to the sum of the reciprocals of the impedances of the single circuit elements. Consequently, if two circuit elements have appreciably different impedances, their overall impedance is controlled by the circuit element of higher impedance if they are in series, and by the circuit element of lower impedance if they are in parallel.

A tBLM can be regarded as a series of slabs with different dielectric properties. When ions flow across each slab following a perturbing AC voltage, they give rise to an ionic current *I*_ion_ that is proportional to the electric field ***E***, according to a proportionality constant σ, called conductivity; the phase shift of *I*_ion_ with respect to the AC voltage equals zero. Ions may also accumulate at the boundary between contiguous dielectric slabs, causing a discontinuity in the electric displacement vector ***D***, which is equal to the electric field ***E*** times the dielectric constant *ε*. Under AC conditions, the accumulation of ions at the boundary of the dielectric slabs varies in time, and so does the electric displacement vector, giving rise to a capacitive current *I*_c_, which is given by the time derivative of ***D***; the phase shift of *I*_c_ with respect to the AC voltage equals −*π*/2. The total current is, therefore, given by the vector sum of the ionic current and the capacitive current. In this respect, each dielectric slab can be simulated by a resistance, accounting for the ionic current, with in parallel a capacitance, accounting for the capacitive current. This parallel combination of a resistance *R* and a capacitance *C* is referred to as a “*RC* mesh”. Accordingly, the impedance spectrum of a tBLM can be simulated by a series of RC meshes.

**Figure 2:**
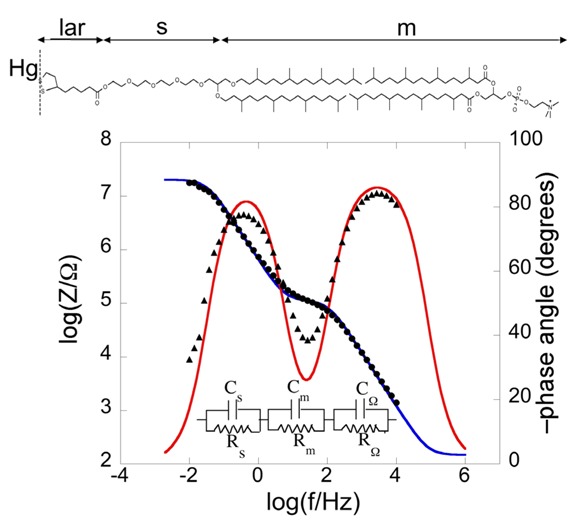
Plot of log|*Z*| (solid circles) and *φ* (solid triangles) against log*f* (Bode plot) for a mercury-supported DPTL/DPhyPC bilayer incorporating gramicidin from its 1 × 10^−7^ M solution in aqueous 0.1 M KCl at −0.60 V *vs.* Ag|AgCl|0.1M KCl. The blue and red curves are the best fit of the equivalent circuit shown in the figure to the impedance spectrum, with *C*_s_ = 9 μF cm^−2^, *R*_s_ = 0.45 MΩ cm^2^, *C*_m_ = 0.65 μF cm^−2^, *R*_m_ = 2.5 kΩ cm^2^, *C*_Ω_ = 0.05 μF cm^−2^ and *R*_Ω_ =3.2 Ω cm^2^. The parameters at the same tBLM in the absence of gramicidin are: *C*_s_ = 5 μF cm^−2^, *R*_s_ = 0.60 MΩ cm^2^, *C*_m_ = 0.85 μF cm^−2^, *R*_m_ = 4.5 MΩ cm^2^, *C*_Ω_ = 0.05 μF cm^−2^ and *R*_Ω_ = 3.2 Ω cm^2^.

Bearing this in mind, let us consider a biomimetic membrane consisting of a thiolipid tethered to the electrode surface, with a lipid monolayer on top of it. As a first approximation, this tBLM can be regarded as consisting of three adjacent slabs: the hydrophilic spacer moiety, the lipid bilayer moiety, and the aqueous solution bathing the lipid bilayer. A simple equivalent circuit commonly employed to interpret the impedance spectrum of a tBLM is shown in the inset of Figure 2; *R*_Ω_, *R*_m_ and *R*_s_ are the resistances of the aqueous electrolyte, the lipid bilayer and the hydrophilic spacer, respectively, whereas *C*_Ω_, *C*_m_ and *C*_s_ are the corresponding capacitances. As an example, we will consider a tBLM consisting of a DPTL monolayer anchored to a mercury electrode, with a DPhyPC monolayer on top of it [24]. The tBLM incorporates gramicidin, a linear neutral pentadecapeptide that spans lipid bilayers by forming a N-terminus-to-N-terminus dimer. The elements of the equivalent circuit are influenced by the movement of K^+^ ions across the lipid bilayer, allowed by the gramicidin channel. The spectrum is displayed on a Bode plot (see Figure 2), namely a plot of both log|*Z*| and phase angle *φ* against log *f*, where |*Z*| is the magnitude of the impedance.

As already stated, the impedance of circuit elements in series is determined by the element with the highest impedance; conversely, the impedance of circuit elements in parallel is determined by the element with the lowest impedance. At the highest frequencies the overall impedance |*Z*| is determined by the resistance *R*_Ω_, because the capacitive impedances, 1/(*ωC*_s_) and 1/(*ωC*_m_), of the *R*_s_*C*_s_ and *R*_m_*C*_m_ meshes, which are lower than the corresponding resistive impedances, are *<< R*_Ω_. At the highest frequencies, |*Z*| is therefore mainly controlled by *R*_Ω_; consequently, it is almost independent of the frequency, whereas the phase angle differs only slightly from the zero value, corresponding to a pure resistive element. With decreasing frequency, 1/(*ωC*_m_) becomes greater than *R*_Ω_, while still remaining lower than *R*_m_, and it is also > 1/(*ωC*_s_), because *C*_s_ is > *C*_m_. Hence, |*Z*| coincides with 1/(*ωC*_m_), and the log|*Z*| *vs.* log *f* plot has a slope equal to −1, while the phase angle tends to −90°, attaining a maximum value of −78° at log (*f*/Hz) = −0.41. With a further decrease in frequency, 1/(*ωC*_m_) becomes comparable with and ultimately greater than *R*_m_, and the log|*Z*| *vs.* log *f* plot tends to become independent of frequency, which would correspond to complete control by *R*_m_. At the same time, *φ* decreases tending to zero. However, before this can occur, a further decrease in frequency makes 1/(*ωC*_s_) >> *R*_m_, causing |*Z*| to coincide with 1/(*ωC*_s_). Hence, the slope of the Bode plot becomes once again equal to −1 and *φ* tends to −90°.

The blue and red curves in Figure 2 are the best fit of the (*R*_s_*C*_s_)(*R*_m_*C*_m_)(*R*_Ω_*C*_Ω_) equivalent circuit to the experimental plot. In spite of its intuitive behavior, the Bode plot is rather featureless. The legend of Figure 2 also reports the resistances and capacitances of the three *RC* meshes for the same tBLM before the addition of gramicidin. It is apparent that the main effect of this peptide consists in decreasing the resistance *R*_m_ of the lipid bilayer by about three orders of magnitude.

Impedance spectra can also be displayed on other types of plots. To justify their use, we must consider that the impedance *Z* of a single *RC* mesh is given by:

*Z*^-1^ = *R*^-1^ - i*ωC* (1)

Writing *Z ≡ Z*′ + i*Z*′′, where *Z*′ and *Z*′′ are the in-phase and quadrature components of the impedance *Z*, and rearranging terms, we obtain:

*Z*′ = *R*/(1+ *ω*^2^*R*^2^C^2^) (a); *Z*″ = *Z*′ *ωRC (b)* (2)

Eliminating *ωRC* from Equations (2a,b) we get:

*Z*″^2^ + *Z*′^2^ - *RZ*′ = 0 → (*Z*′ - *R*/2)^2^ + *Z*″^2^ = (*R*/2)^2^ (3)

Equation (3) yields a semicircle of diameter *R* and center of coordinates (*R*/2,0) on a *Z*′′ *vs.* *Z*′ plot, called “Nyquist plot” [3]. Noting that the maximum of this semicircle is characterized by the equality of *Z*′ and *Z*′′, from Equation (2b) it follows that the angular frequency, *ω*, at this maximum equals the reciprocal of the time constant *RC* of the mesh. In the presence of a series of *RC* meshes, their time constants are often close enough to cause the corresponding semicircles to overlap partially. If the mesh of highest time constant has also a resistance, *R*_x_, much higher than the others, as is often the case for *R*_m_ with respect to *R*_Ω_ and *R*_s_, then the Nyquist plot of the whole impedance spectrum appears as if it were composed of a single well-formed semicircle, *R*_x_ in diameter. The semicircles of the remaining meshes are compressed in a very narrow area close to the origin of the *Z*′′ *vs.* *Z*′ plot, and can be visualized only by enlarging this area. Therefore, the Nyquist plot of the whole spectrum can be conveniently employed if one is interested in pointing out the resistance *R*_x_ of the dielectric slab of highest resistance [3].

To better visualize all semicircles corresponding to the different dielectric slabs composing a tBLM, we have found it convenient to display impedance spectra on a *ωZ*′ *vs.* −*ωZ*′′ plot [3]. Henceforth, this plot will be briefly referred to as a “M plot”, since *ωZ*′ and *ωZ*′′ are the components of the modulus function *M*. A single RC mesh yields a semicircle even in this plot. Thus, if we multiply both members of Equation (3) by *ω*^2^ and we combine the resulting equation with Equation (2b), after simple passages we obtain:

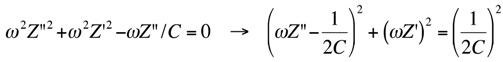
(4)


**Figure 3:**
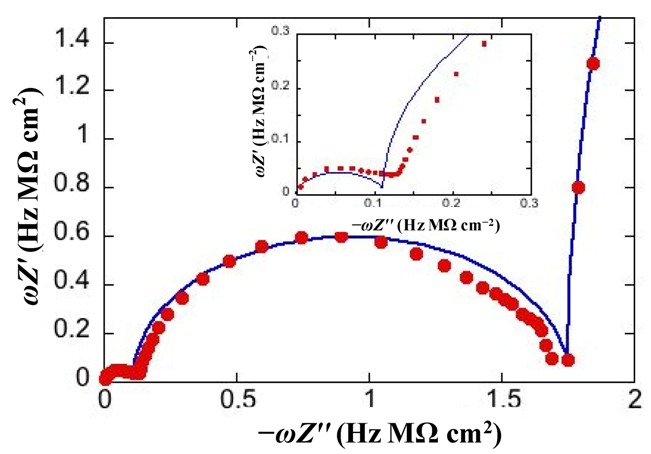
Plot of *ωZ*′ against −*ωZ*′′ for the same system as in Figure 2. The red circles are experimental values and the blue curve is the best fit. The inset shows an enlargement of the distorted semicircle of smallest diameter (and, hence, highest capacitance) ascribed to the hydrophilic spacer.

This is the equation of a semicircle of diameter *C*^−1^ and center of coordinates (2/*C*, 0) on a *ωZ*′ *vs.* −*ωZ*′′ plot. Moreover, *ω* at the maximum of the semicircle is again equal to the reciprocal of the time constant *RC* of the mesh. While *ω* decreases along the positive direction of the abscissa on a Nyquist plot, it increases on a M plot. Therefore, for a series of *RC* meshes, the last semicircle at the right hand side of the M plot is characterized by the lowest time constant. This is, unavoidably, the semicircle simulating the solution that baths the self-assembled film, due to its very low capacitance. Figure 3 shows the M plot relative to the same impedance spectrum that yields the Bode plot in Figure 2. The blue curve is the best fit by the equivalent circuit in the inset of Figure 2, consisting of three *RC* meshes in series. Proceeding along the positive direction of the abscissa, we first find a distorted semicircle of small diameter (and, hence, of high capacitance), which is ascribed to the hydrophilic spacer. The second, larger semicircle is ascribed to the lipid bilayer moiety of the tBLM. Finally, we find the initial portion of a large semicircle ascribable to the aqueous solution that baths the tBLM. With a three-electrode system, the capacitance of the *R*_Ω_*C*_Ω_ mesh yielding this semicircle is that of a thin solution shell around the mercury drop and is very low, amounting to a few tens of nanofarads per square centimeter. The *RC* mesh of the aqueous solution does not depend on the architecture of the tBLM. Hence, the corresponding semicircle is usually excluded almost completely from a M plot to better visualize the contribution from the other semicircles. The M plot permits the agreement between an experimental impedance spectrum and the corresponding fit by a series of RC meshes to be verified in greater detail than on a Bode or Nyquist plot.

Under certain circumstances, EIS reveals further dielectric slabs in addition to those normally expected in Hg-supported tBLMs (*i.e.*, spacer, lipid bilayer, aqueous solution) and SAMs (hydrocarbon tails, polar heads, aqueous solution). This is the case when a mercury-supported SAM consists of a mixture of lipids forming gel phase microdomains and “lipid rafts”. Lipid rafts are microdomains, composed of glycolipids, sphingolipids and cholesterol (Chol), which modulate the membrane function in eukaryotic cells and are involved in cell signaling and molecular trafficking [25,26,27]. Sphingolipids and glycolipids have a melting temperature higher than room temperature, because their hydrocarbon tails are saturated, and therefore, chain length being equal, they are longer than the unsaturated phospholipids that constitute the matrix surrounding the gel-phase microdomains and the rafts. In the absence of Chol, sphingolipids and saturated phospholipids form gel phase microdomains, also called “solid ordered” microdomains, which are anisotropic, tightly packed and have limited lateral mobility. Conversely, the matrix is isotropic, less densely packed and has a high degree of lateral mobility, such that it is also defined as “liquid disordered”. Insertion of Chol in the gel phase microdomains increases lateral mobility, removes anisotropy and converts them into lipid rafts. Lipid rafts are also defined as “liquid ordered” because they are “liquid”, thanks to a sufficient lateral mobility, but “ordered” because more tightly packed than the liquid disordered matrix. In the presence of microdomains, which are thicker than the surrounding matrix, a mercury-supported SAM can be regarded as consisting of a series of slabs with different dielectric properties [6].

**Figure 4:**
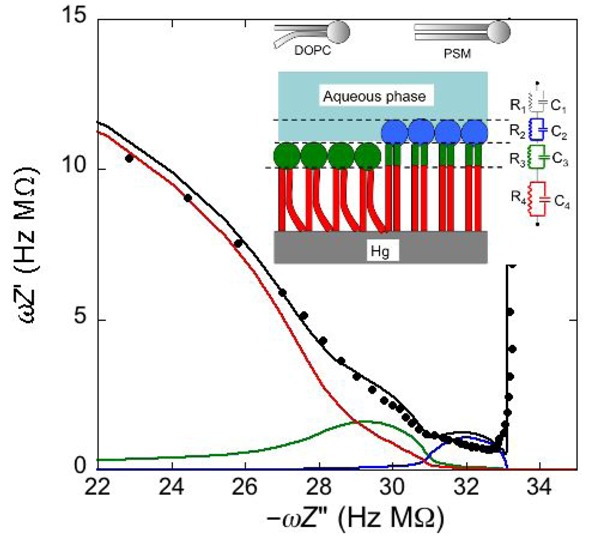
Plot of *ωZ*′ against −*ωZ*′′ for a Hg-supported monolayer of a DOPC/PSM (50:50) mixture in aqueous 0.1 M KCl at −0.55 V. Black circles are experimental points and the black curve is the best fit to these points obtained with a series of four *RC* meshes. The blue, green and red curves are the single *ωZ*′ contributions to this fit ascribable to the polar heads of PSM plus water molecules (i), the polar heads of DOPC plus the upper portion of the PSM hydrocarbon tails; (ii), and the hydrocarbon tail region; (iii), respectively. The capacitances and resistances of the corresponding *RC* meshes are: *C*_2_ = 33 μF cm^−2^, *R*_2_ = 0.28 kΩ cm^2^, *C*_3_ = 20 μF cm^−2^, *R*_3_ = 18 kΩ cm^2^, *C*_4_ = 2.8 μF cm^−2^, *R*_4_ = 3.5 MΩ cm^2^, where the subscripts 2, 3 and 4 refer to the dielectric slabs (i), (ii) and (iii).

Consider a mercury-supported SAM of a lipid mixture of palmitoylsphyngomyelin (PSM) and DOPC forming gel phase microdomains. The outer slab consists of the polar heads of PSM, mixed with water molecules. Below this, we find a slab consisting of the polar heads of DOPC and of the upper portion of the hydrocarbon tails of PSM, and then a final slab consisting of the hydrocarbon tails of both lipids. This lipid SAM can be simulated by three *RC* meshes in series, representing the above three slabs, plus an additional *RC* mesh representing the aqueous solution bathing the SAM. On a M plot, each *RC* mesh is represented by a semicircle distorted by the neighboring semicircles, as shown in Figure 4. The diameter of each semicircle measures the reciprocal of the capacitance of the corresponding *RC* mesh.

**Figure 5:**
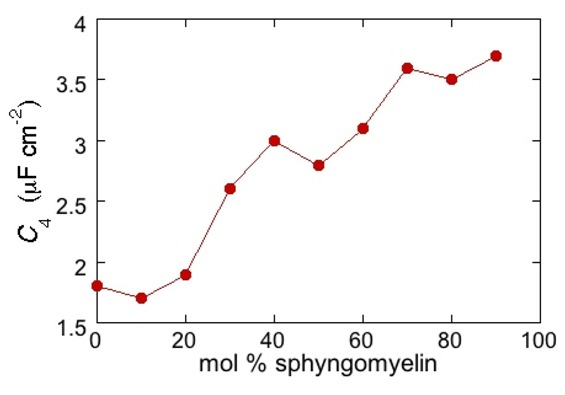
Plot of the capacitance *C*_4_ of the hydrocarbon tail region for a Hg-supported DOPC/PSM (50:50) monolayer in aqueous 0.1 M KCl at −0.55 V, as a function of mol% PSM.

Proceeding from right to left and disregarding the *R*_Ω_*C*_Ω_ mesh, we observe a distorted semicircle ascribable to the PSM polar heads, a further distorted semicircle ascribable to the polar heads of DOPC and a final semicircle of larger diameter, and hence lower capacitance, ascribable to all the hydrocarbon tails. The capacitance *C*_4_ of this semicircle is particularly informative. If we plot it as a function of the mole fraction of PSM in the binary mixture with DOPC, we observe two rather abrupt increases in capacitance, as shown in Figure 5.

From a comparison with the binary DOPC-PSM phase diagram reported in [28] on the basis of time-resolved Fluorescence Resonance Energy Transfer (FRET) measurements, it is apparent that the first increase corresponds to the transition from the liquid disordered phase to a mixture of liquid disordered and solid ordered phase, whereas the second increase corresponds to the passage from this mixture to the sole solid ordered phase. This behavior can be explained by the mismatch between the anisotropic gel phase microdomains and the surrounding liquid disordered matrix offering a preferential pathway to the ions that move back and forth across the lipid monolayer, following the AC voltage. This causes an increase in differential capacitance, which is roughly proportional to the total length of the edges of the gel phase microdomains.

**Figure 6:**
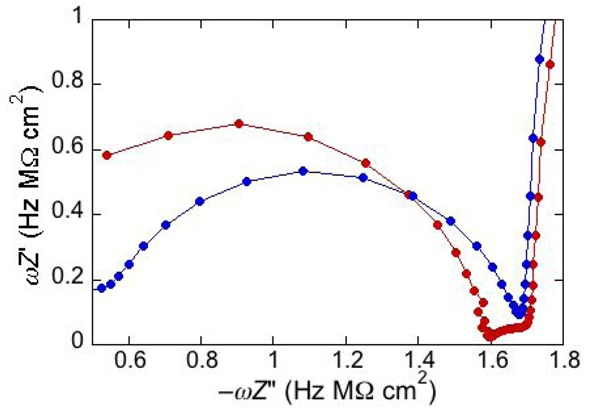
Plot of *ωZ*′ against −*ωZ*′′ for a DPTL/(DOPC:PSM:Chol) tBLM at −0.60 V in an aqueous solution of 0.1 M KCl and 1.4 × 10^−7^ M melittin (red curve) and for a DPTL/DOPC tBLM in the same solution (blue curve).

An unusual additional dielectric slab is also monitored at a tBLM whose distal monolayer consists of a raft-forming mixture (DOPC:PSM:Chol) in contact with a solution of the antimicrobial peptide melittin [29]. The M plot at −0.60 V for a DPTL/(DOPC:PSM:Chol) tBLM immersed in a solution of melittin (red curve in Figure 6) differs from that for a DPTL/DOPC tBLM under otherwise identical conditions (blue curve in Figure 6) by the presence of a small partially fused semicircle; this is interposed between a semicircle ascribable to the lipid bilayer, at lower frequencies, and the almost linear initial portion of a very large semicircle, ascribable to the aqueous phase, at higher frequencies. Interestingly, this small additional semicircle is only observed at potentials positive of −0.80 V. It was simulated by adding a further *RC* mesh to the equivalent circuit used for the fitting of the impedance spectrum [29]. The in phase admittance of this further *RC* mesh attains values more that two orders of magnitude higher than those of the corresponding lipid bilayer moiety; its contribution practically disappears at potentials negative of about −0.85 V, at which the conductance of the lipid bilayer moiety starts increasing due to the opening of the voltage-gated channel of melittin. The dielectric slab responsible for this additional *RC* mesh is explained by an accumulation of melittin molecules on top of the liquid-ordered microdomains (the lipid rafts), where lipid lateral movements are reduced by higher cohesive properties. These melittin molecules, lying flat on top of the polar heads of these microdomains, may form local “carpets” which, intercalated with water molecules, are detected by EIS as a dielectric slab of high conductance. This interpretation may also explain the disappearance of the features of this additional dielectric slab at the negative applied potentials at which the conductance of the lipid bilayer moiety undergoes an abrupt increase, due to insertion of the melittin molecules into the bilayer with channel formation. The insertion is likely to take place at the boundaries of lipid rafts and of possible gel phase microdomains, where the mismatch between these microdomains and the surrounding liquid-disordered matrix may favor the penetration of the melittin molecules into the lipid bilayer.

## 3. Potential Step Chronocoulometry

**Figure 7:**
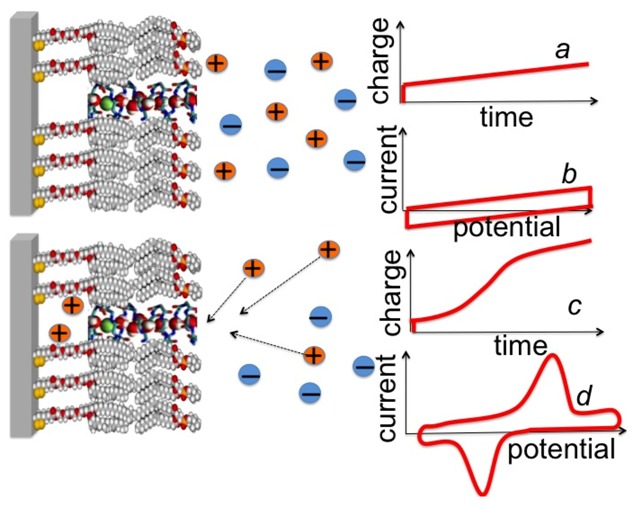
Schematic pictures of a DPTL/DOPC tBLM incorporating a gramicidin channel in the closed state (**top left**) and in the open state (**bottom left**). The typical shape of charge *vs.* time and current *vs.* potential curves at a closed ion channel (or in the absence of ion channels) and at an open ion channel is depicted on the right side of the corresponding schematic pictures.

Potential-step chronocoulometry consists in subjecting the electrochemical system under investigation to a potential jump from an initial value *E*_i_ to a final value *E*_f_ and in recording the charge *Q*(*t*) that flows as a consequence of this jump as a function of time. When applied to a mercury-supported DPTL/lipid tBLM in the absence of channel-forming peptides, the charge transient triggered by a potential jump is characterized exclusively by an initial flux of purely capacitive charge, lasting less than one millisecond; at sufficiently negative *E*_f_ values, this is followed by a linear increase of charge in time, due to a constant small reduction current ascribable to electroactive trace impurities, as schematically depicted in Figure 7, curve *a*.

Upon incorporating cation-selective or nonselective ion channels, either ohmic or voltage-gated, a potential jump from an initial potential *E*_i_, at which the ion channel is closed or not yet formed, to a sufficiently negative final potential *E*_f_, at which it is open, causes an ion flux along the channel into the tetraethyleneoxy (TEO) hydrophilic spacer. In particular, ohmic channels allow an ion flux into the TEO spacer in the proximity of a transmembrane potential, *ϕ*_trans_, equal to zero, while voltage-gated channels show this behavior only at some negative transmembrane potential. The ion flux is revealed by a negative charge that adds to the background charge recorded in the absence of channels. This negative charge is due to electrons that flow along the external circuit and accumulate on the metal surface, to maintain the electroneutrality of the whole electrified interface interposed between the bulk metal and the bulk aqueous solution. The electron charge density σ_M_ accumulating on the metal surface is practically equal in magnitude and opposite in sign to the cation charge density accumulating in the hydrophilic spacer. In fact, the only other charge density located within the electrified interface is that present in the diffuse layer adjacent to the tBLM, which is much smaller than the other two [30]. As the final potential *E*_f_ is made progressively more negative, an *E*_f_ value is ultimately attained at which the TEO spacer is completely saturated by the cations of the electrolyte. This gives rise to a charge step with a well-defined plateau, which adds to the background charge, as shown schematically in Figure 7, curve *c*. In the case of potassium ions, the height of this charge step ranges from −45 to −50 μC cm^−2^ and corresponds to the maximum charge of potassium ions that can be accommodated in the TEO spacer, as verified for different ion channels, such as gramicidin [24], melittin [31], monazomycin [31], distinctin [17] and alamethicin [17]. 

Let us first consider the behavior of the antibiotic peptide gramicidin, which forms an ohmic ion channel selective toward small monovalent cations such as alkali metal ions and thallous ion. The channel consists of a single N-terminus-to-N-terminus dimer, probably via the formation of six intramolecular hydrogen bonds [32,33]. In fact, the hydrocarbon-tail portion of a lipid bilayer, whose length ranges from 30 to 40 Å, can only be spanned by two aligned gramicidin molecules, which form a channel about 25 to 30 Å long [33]. The three tryptophan residues located at the C-terminus of the gramicidin molecule interact with the polar heads of the bilayer, thanks to their H-bonding capability and favorable electrostatic interactions. As distinct from the majority of antibiotic peptides, which form *α*-helices, gramicidin forms a 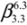 -helix, whose lumen is large enough to allow the passage of partially desolvated monovalent cations. Conversely, the lumen of *α*-helices is too narrow to allow the passage of ions, no matter how small they may be. Hence, *α*-helices may form ion channels by aggregating into bundles. 

**Figure 8:**
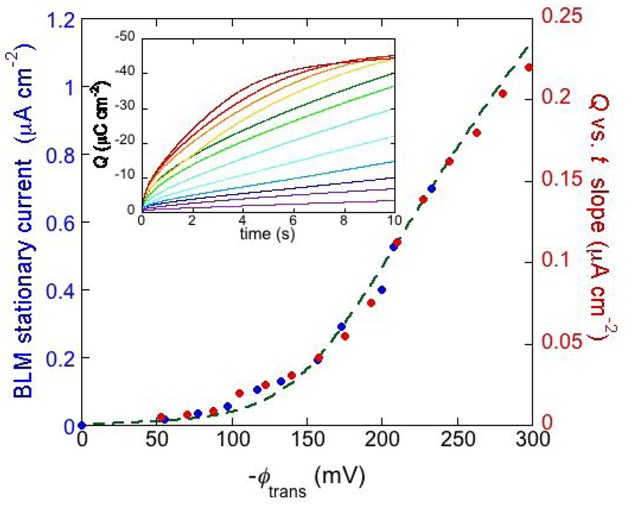
The inset shows a number of charge *vs.* time curves following potential jumps from −0.25 V towards progressively more negative final potentials at a DPTL/DPhyPC tBLM incorporating gramicidin from its 1.0 × 10^−7^ M solution in aqueous 0.1 M KCl. The red circles are values of the slope of the linear portion of the charge *vs.* time curves, plotted against the transmembrane potential *ϕ*_trans_. The blue circles are values of the stationary current at a conventional BLM incorporating gramicidin A from its 2 × 10^−11^ M solution in aqueous 1 M NaCl, plotted against *ϕ*_trans_ (from Figure 2 in Ref. [34]). The dashed green curve was calculated as outlined in the text.

The inset of Figure 8 shows a number of charge *vs.* time curves following a series of potential jumps from a fixed initial potential *E*_i_ = −0.25 V to progressively more negative final potentials, *E*_f_, at a DPTL/DPhyPC tBLM incorporating gramicidin from its 1.0 × 10^−7^ M solution in aqueous 0.1 M KCl [24]. Before each potential jump, the electrode is kept at *E*_i_ for 5 min. During this rest time, potassium ions are almost completely expelled from the TEO spacer by electrostatic repulsion. The higher the negative potential jump, the larger the amount of K^+^ ions moved into the TEO spacer along the gramicidin channels. Ultimately, the TEO spacer is saturated with K^+^ ions, and the charge attains a constant limiting value of about −45 μC cm^−2^. The plateau of the charge *vs.* time curves is attained at shorter times the more negative *E*_f_ is. Before tending to this limiting value, the curves of the charge *Q* against the time *t* exhibit a roughly linear section, whose slope measures the “stationary current” that would be maintained if there were no limitations to K^+^ diffusion on the metal side of the lipid bilayer moiety of the tBLM, as in the case of traditional BLMs. The slope of the linear section of the *Q vs. t* curves is plotted against the transmembrane potential *ϕ*_trans_ in Figure 8 (red circles). The blue circles are stationary currents obtained directly at a conventional BLM [34]. It is evident that the stationary current increases with |*ϕ*_trans_| much more than linearly. This behavior is in apparent contrast with the fact that the two monomers forming the dimeric channel have appreciable dipole moments with opposite orientations. Hence, if the association constant *k*_a_ for dimer formation were independent of potential, then the maximum stability of the dimeric channel would be attained at zero transmembrane potential, and a potential shift in any of the two directions would favor one monomeric orientation at the expense of the other, destabilizing the dimer. To explain the experimental behavior we must assume that the stability of the dimeric channels increases with an increase in the ion flux along them. In other words, the more rapid the single-file motion of ions along gramicidin channels is, the more it prevents their dissociation. In fact, when the time elapsed between the passage of two consecutive cations through the junction between the two monomers forming the conducting dimer starts to become comparable with, and ultimately shorter than, the time required for the mismatch between the two monomers with dimer dissociation, the latter event is expected to become less probable. To account for this effect, the association constant for dimer formation was multiplied by a dimensionless quantity proportional to the continuously growing ion flux, and a constant damping factor was introduced to moderate the increase of *k*_a_, which otherwise would be exceedingly high [24]. The dashed curve in Figure 8 was calculated by this “feedback” procedure.

**Figure 9:**
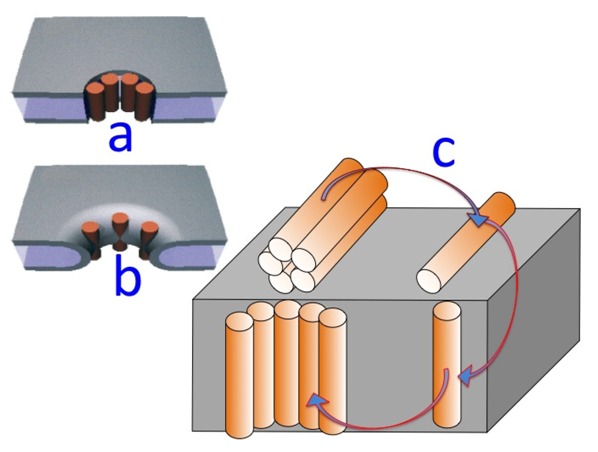
Schematic pictures of a barrel-stave model (**a**); of a toroidal model (**b**); and of the gradual passage from a “flat cluster” to an “embedded cluster” (*i.e.*, an ion channel) via a “flat monomer” and an “embedded monomer” (**c**).

*α*-Helical peptides capable of permeating membranes are normally “amphipathic”, namely they have one side of the helix relatively hydrophilic by the presence of residues with hydrophilic and/or charged side chains, whereas the other side is hydrophobic. They may permeate a membrane by two main alternative mechanisms, the “barrel stave” and the “carpet” mechanism [35]. According to the first mechanism, the peptide monomers associate to form a bundle inside the membrane, turning their hydrophilic side toward the interior of the bundle and their hydrophobic side toward its exterior, where the latter side interacts attractively with the hydrocarbon tails of the surrounding lipid matrix. The central lumen of the bundle, lined by the hydrophilic side chains, constitutes an ion channel, like a barrel made of helical peptides as staves (see Figure 9a). A variant of the barrel stave model is the “toroidal model” [36], in which the peptides are associated with the lipid polar heads even when they are perpendicularly inserted into the lipid bilayer. In forming such a pore the lipid molecules around the pore bend continuously along its side up to assuming a horizontal orientation at half height of the pore, thus intercalating their polar heads between the vertical peptide molecules (see Figure 9b). In this way, the pore is lined by both the peptides and the lipid polar heads. According to the carpet model, the peptide molecules remain in contact with the polar heads of the lipid bilayer during the whole process of membrane permeation and do not insert into the hydrophobic core of the membrane. Thus, they do not need necessarily to adopt an amphipathic *α*-helical structure. Membrane permeation takes place only if there is a high local concentration of membrane-bound peptides, with their hydrophilic side facing the lipid polar heads and/or the aqueous phase. Ultimately, the peptides disintegrate the membrane by disrupting the bilayer curvature. It has been proposed that this disruption involves a detergent-like micellization, whereby lipid micelles are enveloped by a peptide layer and stripped from the lipid bilayer, forming transient holes in the membrane.

Potential step chronocoulometry provides useful information on the mechanism of membrane permeabilization by amphipathic *α*-helical peptides. In particular, it may monitor the formation of ion channels by the barrel stave or toroidal mechanism via the cooperativity in the peptide aggregation process. Moreover, it allows an approximate estimate of the time required for ion channel formation and for its disruption. Finally, it permits one to verify whether the peptide is initially adsorbed on the membrane surface as a monomer or an oligomer and whether monomers adsorbed flat on the membrane surface have a tendency to form aggregates there. Potential step chronocoulometry allows the achievement of these results by carrying out rapid potential jumps from an initial value *E*_i_ at which an ion channel is closed or not yet formed to a final, more negative value at which it is formed and open, thus saturating the spacer with small inorganic cations.

With peptides that form ion channels by aggregation of helices turning the hydrophilic side toward the lumen of the channel, the charge transients following a potential jump are often characterized by a sigmoidal shape. This shape can be explained on the basis of a general kinetic model that accounts for the potential-dependent penetration of adsorbed monomeric molecules into the lipid bilayer, followed by their aggregation with channel formation by a mechanism of nucleation and growth. This mechanism involves a short induction period that is responsible for an initial lower portion of the charge *vs.* time curve with the concavity turned upwards; since the upper portion turns the concavity downwards, tending to a maximum saturation value, this behavior leads to a sigmoidal charge transient, which is exhibited by the antimicrobial peptides melittin [31] and trichogin GA IV [37]. With several peptides, the curve of the charge *Q* against the time *t* also exhibits an initial horizontal portion that may last for times as long as 100 s, upon subtracting the capacitive contribution recorded in the absence of the peptide. This is the case with the macrocyclic polyol lactone monazomycin [31], and the peptides alamethicin [17], distinctin [17] and dermcidin [38]. This long “foot” of the *Q vs. t* curve can be explained by the presence of peptide oligomers initially adsorbed flat on top of the lipid bilayer; these oligomers must undergo disaggregation before penetrating the hydrocarbon tail region.

The overall process of oligomer disaggregation into monomers, their penetration into the bilayer and re-aggregation with ion channel formation can be interpreted on the basis of a mechanism involving a kinetics of nucleation and growth. According to this mechanism, the potential jump induces the disruption of any clusters adsorbed on top of a tBLM (henceforth referred to as “flat clusters”), the penetration of the resulting “flat monomers” into the lipid bilayer and the aggregation of the monomers incorporated in the lipid bilayer (henceforth referred to as the “embedded monomers”) into channel-forming “embedded clusters” (see Figure 9c for a scheme of this process). The kinetic process of disruption of flat clusters is treated as a nucleation and growth of holes within the clusters. By “nucleation” of holes, we mean the quasi-reversible detachment of an initial number of flat monomers from a flat cluster and their random intercalation with the water molecules on top of the tBLM. In other words, a hole is just a flat monomer that, upon detachment from a flat cluster, leaves behind a hole in the cluster. These flat monomers are considered to detach from a flat cluster and to re-aggregate to it in a quasi-reversible manner, until the number of nearest-neighboring holes in the cluster attains a critical value (*n*) beyond which this number increases irreversibly up to complete disruption of the flat cluster. This critical number of nearest-neighboring holes constitutes the “nucleus”. Nucleation is followed by the irreversible “growth” of the nuclei. These are formed progressively in time, with a nucleation rate constant (*k*_h,N_); each one grows irrespective of the others, with a given rate of radial growth (*v_h_*_,*R*_). Possible overlapping of the growing nuclei is avoided by using a formalism introduced by Avrami [39]. It can be shown that the kinetics of nucleation and growth of holes is expressed by a single adjustable parameter, *i.e.*, *k*_h,N_ *v*_h,R_^2^ [31]. Disruption of flat clusters starts only when the interfacial electric field becomes high enough to drag the few flat monomers in equilibrium with flat clusters into the lipid bilayer, converting them into embedded monomers. We define by *p* the probability of penetration of monomers into the lipid bilayer; this is a dimensionless parameter that measures the interfacial electric field strength and increases as the potential difference across the lipid bilayer (*i.e.*, the transmembrane potential) becomes progressively more negative, until it reaches its limiting unit value. The disruption of flat clusters is necessary because they are expected to have the hydrophilic and/or charged groups turned toward the aqueous phase and the hydrophobic groups turned toward the interior of the clusters. Conversely, the embedded monomers resulting from disruption of the flat clusters tend to aggregate with the hydrophilic groups turned toward the interior of the embedded cluster and the hydrophobic groups turned toward its exterior. This gives rise to embedded clusters with a lumen lined by hydrophilic and/or charged groups, namely “ion channels”. The formation of ion channels is again assumed to proceed by nucleation of the embedded monomers and growth of the resulting nuclei and involves an additional shorter induction period, which is responsible for the initial upward concave portion of the sigmoidal charge step. The number of embedded monomers forming the critical nucleus is assumed to be equal to that, *n*, for the nuclei of holes, for simplicity. The kinetics of the nucleation and growth process yielding ion channels is expressed by the single adjustable parameter *k*_N_ *k*_R_^2^, where *k*_N_ is the rate constant of nucleation of the embedded monomers and *k*_R_ is the rate constant of radial growth of the resulting nuclei [31]. The ions moving across the lipid bilayer moiety of the tBLM accumulate inside the hydrophilic spacer, spreading radially from the mouth of each newly formed channel, until they completely saturate it. When this condition is fulfilled, the charge transient attains a plateau that depends exclusively on the spaciousness of the spacer. The model must, therefore, associate the nucleation and growth process yielding ion channels to the concomitant radial diffusion of the translocating ions into the hydrophilic spacer. This is accomplished by assuming that the formation of each newly formed channel starts a radial diffusion of ions from its mouth [31]. The kinetics of this radial diffusion of ions, leading to complete saturation of the hydrophilic spacer, is expressed by a single parameter, namely *k*_d,R_; this measures the time derivative of the square of the radius of the circular area covered by the ions flowing from the mouth of each single channel and diffusing radially into the hydrophilic spacer. It can be shown that *k*_d,R_ is approximately constant [31,40].

**Figure 10:**
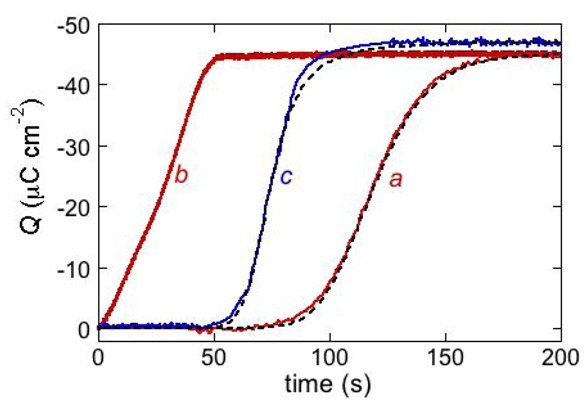
The red curves *a* and *b* are charge transients following potential jumps from −0.30 V to the −1.00 V at a DPTL/DOPC tBLM immersed in aqueous 0.1 M KCl containing 0.4 μM distinctin. Curve *a* was obtained by the pristine potential jump; curve *b* was obtained by a second potential jump after a rest time of 30 s at −0.30 V. The dashed curve *a* was calculated using the parameters *θ*_0_ = 0.1, *n* = 2, *k*_h,N_ *v*_h,R_^2^ = 1 × 10^−4^ s^−3^, k_N_ *k*_R_^2^ = 4 × 10^3^ s^−3^, *k_d_*_,R_ = 7 × 10^−3^ cm^2^ s^−1^ and *p* = 0.1. The parameter *θ*_0_ denotes the fraction of the whole surface of the tBLM initially covered by flat clusters. The blue curve *c* is a charge transient following a potential jump from −0.30 V to −0.65 V at a DPTL/DOPC tBLM immersed in aqueous 0.1 M KCl containing 0.4 μM alamethicin. The corresponding dashed curve was calculated using the parameters *θ*_0_ = 0.4, *n* = 2, *k*_h,N_ *v*_h,R_^2^ = 1 × 10^−6^ s^−3^, k_N_ *k*_R_^2^ = 4 × 10^4^ s^−3^, *k_d_*_,R_ = 7 ×10^−3^ cm^2^s^−1^ and *p* = 1.

The above behavior is exemplified by the *Q vs. t* curve of the antimicrobial peptide distinctin [17] (curve *a* in Figure 10). It was obtained at a DPTL/DOPC tBLM in a solution of 0.1 M KCl and 0.4 μM distinctin by stepping the applied potential from *E*_i_ = −0.30 V to *E*_f_ = −1.00 V. The rest time at *E*_i_ before this potential jump was fixed at 30 s. The charge involved in the sigmoidal step amounts to about −45 μC cm^−2^ and corresponds to complete saturation of the hydrophilic spacer by potassium ions. If the potential jump yielding the sigmoidal charge step was repeated after a rest time of 30 s at *E*_i_, a much steeper charge step of the same height and almost lacking the sigmoidal shape was obtained (curve *b* in Figure 10). This indicates that a rest time of 30 s at *E*_i_ is not sufficient to dismantle the ion channels formed at *E*_f_. If the rest time at *E*_i_ was of only 3 s, the charge step was steeper and smaller than in the previous case. This proves that a time period of 3 s at *E*_i_ is not sufficient to expel all the positive ions from the hydrophilic spacer.

If melittin is incorporated in a tBLM from its 1.4 × 10^−7^ M aqueous solution and a potential jump is performed immediately afterwards, the resulting sigmoidal charge transient does not show a long foot and can be fitted by the model previously described, in which the initial stage of oligomer disaggregation is skipped. However, if the tBLM is kept in the presence of the melittin solution for a long period of time, say 15 min, at a potential at which the melittin molecules are adsorbed flat on top of the lipid bilayer, an identical potential jump yields a charge transient with a long foot [31]. This indicates that the melittin aqueous solution contains mainly monomers that tend slowly to form flat clusters when in contact with the membrane surface at an applied potential at which ion-channel formation does not occur.

## 4. Cyclic Voltammetry

The cyclic voltammetry technique consists in scanning the applied potential at a constant scan rate from an initial to a final value and in then inverting the potential scan; this potential cycling can be repeated several times during a single experiment. The current is plotted *vs.* the applied potential to yield the cyclic voltammetry trace. When applied to a Hg-supported tBLM in the absence of channel-forming peptides, the negative-going scan gives rise to a roughly constant negative capacitive current, due to a gradual accumulation of electrons on the metal surface and to a parallel accumulation of positive ions in the diffuse layer adjacent to the tBLM. In fact, the current density *j* is given by d*Q*/d*t* = (d*Q*/d*E*) (d*E*/d*t*) = *Cv*, where *Q* is the capacitive charge density and *v* is the scan rate. The current also includes a resistive contribution due to the non-infinite resistance of the lipid bilayer moiety, which imparts a slight tilt to the current trace. The reverse positive-going cyclic voltammetry scan yields a current with the same resistive contribution but an opposite capacitive contribution with respect to the zero-current axis. This results in a trace whose shape is similar to that of a tilted rectangle (see curve *b* in Figure 7). Under these conditions, the vertical distance between the negative and the positive voltage scan is clearly equal to 2*Cv*. 

In the presence of an ion channel, the negative-going scan yields a negative current peak that adds to the background current, as soon as the appropriate transmembrane potential value is attained (see curve *d* in Figure 7). This peak is due to cation inflow in the spacer and/or anion outflow; in the case of K^+^ inflow, integration of the peak current over time yields a charge of −45/−50 μC cm^−2^, corresponding to spacer saturation by this univalent cation. Under steady-state conditions, the positive-going scan yields a positive current whose integration must necessarily provide a charge equal and opposite to that obtained by integration of the negative current. As a rule, cyclic voltammetry curves are not controlled by the diffusion of the permeating ions in the aqueous solution towards or away from the tBLM surface, but rather by the rate at which the ions overcome the potential energy barrier represented by the lipid bilayer interposed between the aqueous solution and the hydrophilic spacer. Differently stated, the concentration of a permeating ion in direct contact with the external surface of the tBLM does not change to a detectable extent with respect to its bulk value during the functional activity of an ion channel. Hence, cyclic voltammetry provides useful information on the kinetics of inflow and outflow of permeating ions, also by varying the scan rate. In particular, it allows a distinction to be made between ohmic and voltage-gated ion channels.

**Figure 11:**
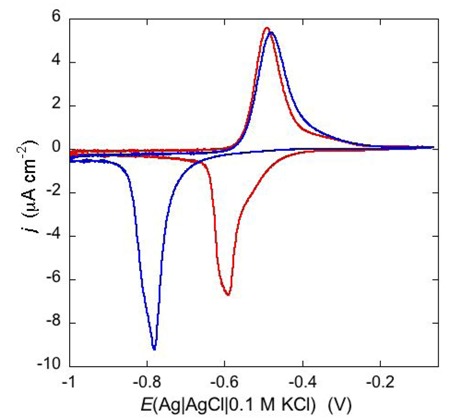
Cyclic voltammograms of a DPTL/DOPC tBLM in aqueous 0.1 M KCl containing 0.3 μM gramicidin, at a scan rate of 10 mV s^−1^, before (blue curve) and after stabilization (red curve).

An example of ohmic ion channel is provided by gramicidin. Figure 11 shows the cyclic voltammogram of a DPTL/DOPC tBLM incorporating gramicidin from its 0.3 μM solution in aqueous 0.1 M KCl [37]. On a freshly prepared tBLM the negative peak lies at about −0.77 V while the positive one lies at −0.47 V. Multiple voltammetric scans shift the negative peak in the positive direction while leaving the positive peak unchanged, until eventually the midpoint potential between the negative and positive peaks comes to practically coincide with a value, −0.52 V, very close to zero transmembrane potential. The shift of the negative peak is due to progressive gramicidin incorporation, which results in an increase in the rate of K^+^ inflow into the hydrophilic spacer. On the other hand, the rate of K^+^ outflow is unaffected by the amount of incorporated gramicidin. The shape of the negative peak is very close to that obtained from the positive peak by capsizing the positive half-plane on the negative one.

**Figure 12:**
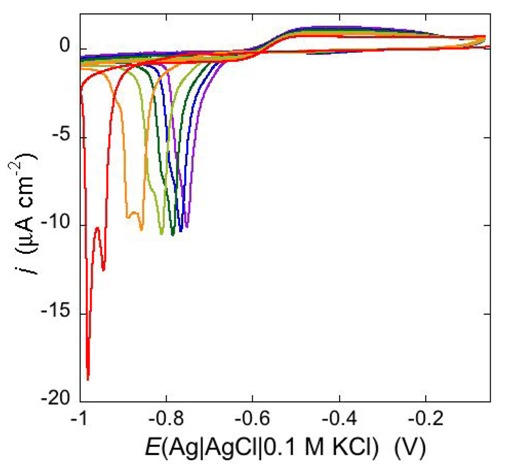
Cyclic voltammograms of a freshly prepared DPTL/DOPC tBLM immersed in aqueous 0.1 M KCl containing 1 μM TCG. Proceeding from left to right, the cyclic voltammograms were obtained at a scan rate of 10 mV s^−1^ after 1, 3, 6, 9, 12 and 15 cycles from the instant of the addition of the peptide.

An altogether different behavior is shown by the peptaibol trichogin GA IV (TCG) [37]. At a scan rate of 10 mV s^−1^, the first negative-going potential scan at a freshly prepared DPTL/DOPC tBLM incorporating TCG from its 1 μM aqueous solution shows two partially overlapping negative current peaks at about −0.95/−1.00 V (see Figure 12). As distinct from the negative-going current, the positive-going one is relatively flat and exhibits a rounded hump at about −0.50 V. Repeated scanning causes the two negative peaks to shift gradually toward less negative potentials and to merge eventually into a single current peak. Current stabilization is attained when the negative current peak is at −0.75/−0.70 V. This positive shift, recorded on the same mercury drop, is ascribed to a progressive incorporation of peptide molecules into the lipid bilayer of the tBLM and/or to a change in the orientation or degree of aggregation of TCG molecules already present in the lipid bilayer. Integration of the area under the negative peak (or peaks) yields a charge density σ_M_ on the metal of about −45 μC cm^−2^ at all scan rates from 5 to 50 mV s^−1^ and at all TCG concentrations from 1 to 5 μM. This implies that the negative-going voltage scan fills the spacer completely with K^+^ ions, while the positive-going one expels them from the spacer into the bulk aqueous phase, in order to maintain steady-state conditions. The charge involved in the positive-going scan cannot be accurately measured because of the flatness of the cyclic voltammogram at potentials positive of −0.60 V. It should be noted that no negative peak is observed on a freshly prepared tBLM incorporating TCG if the voltage scan does not cover potentials more negative than about −0.85 V. In view of the correspondence between the applied potential *E* and the transmembrane potential *ϕ*_trans_, the latter *E* value corresponds to a *ϕ*_trans_ value of about −240 mV, which is outside the range of physiological transmembrane potentials. Conversely, after its incorporation, TCG permeabilizes the lipid bilayer stably at transmembrane potentials of −80/−90 mV, and its effect is detectable with EIS even at *ϕ*_trans_ ≅ −30 mV. These are physiological transmembrane potentials, although their relatively high values suggest a voltage-gated behavior of TCG, analogous to that exhibited by the more widely known and much more extensively investigated peptaibol alamethicin [41,42]. In fact, the cyclic voltammogram of alamethicin incorporated in the tBLM is similar to that of TCG, with a negative sharp current peak and a relatively flat positive current.

When compared with the cyclic voltammogram of the ohmic ion channel gramicidin, the highly asymmetric cyclic voltammogram of TCG can be regarded as typical of a voltage-gated channel, triggered by a trans-negative transmembrane potential. The fact that the first negative-going scan at a freshly prepared tBLM in the presence of TCG does not give rise to any ionic inflow in the hydrophilic spacer for *E* values less negative than −0.85 V indicates that TCG is inactive over this potential range. There is a general consensus [43,44] that this inactivity is associated with an orientation of the helical TCG molecules parallel to the membrane surface. After a single cyclic voltammogram between −0.05 and −1.00 V, a nonzero flat positive current is recorded, with the charge under this current necessarily equal and opposite to that under the negative current peak. This positive current is present at potentials much more positive than those at which TCG starts to be active during the pristine negative-going scan, although the ion flow is slower than the opposite flow at the negative potentials at which the negative peak is recorded. This indicates that the TCG helices do not recover immediately the original orientation parallel to the membrane surface, but assume some intermediate conformation allowing an ion flow, albeit slower than the opposite flow. It is also possible that they do not change conformation but exhibit “sidedness” within the lipid bilayer, such as to favor a higher ion flow in the negative direction than in the positive one.

**Figure 13:**
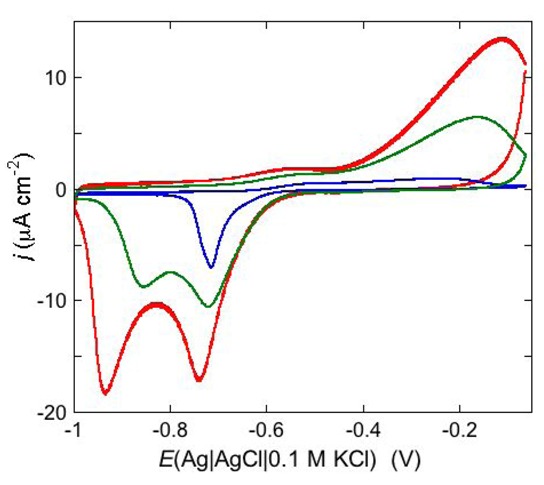
Cyclic voltammograms of a DPTL/DOPC tBLM stabilized in aqueous 0.1 M KCl containing 1 μM TCG. In the order of increasing height, the scan rates are 10, 100 and 200 mV s^−1^.

Under certain circumstances, the dependence of the shape of a cyclic voltammogram upon the scan rate may reveal additional features of the functional activity of ion channels. Thus, an increase of the scan rate from 10 to 200 mV s^−1^ causes the negative peak at a DPTL/DOPC tBLM incorporating TCG to split into two peaks, as shown in Figure 13; while the less negative peak maintains a peak potential close to that of the single peak as recorded at 10 mV s^−1^, the more negative peak shifts markedly in the negative direction with an increase in the scan rate. This behavior can be tentatively explained by a TCG ion channel consisting of a dimer of two aligned TCG helices, in view of the TCG molecule being too short to span a lipid bilayer. At a scan rate of 10 mV s^−1^, all TCG dimers have sufficient time to aggregate with ion channel formation, yielding a single peak. On the other hand, at higher scan rates not all dimers succeed in aggregating over the potential range of the single peak; the unclustered dimers will then induce an ion flux at more negative potentials, yielding the more negative voltammetric peak. Evidence of a TCG dimer having two N-terminuses in close contact and rotating like a solid cylinder was recently provided by a three-pulse stimulated electron spin echo spectrum of spin labeled TCG [44]. The authors hypothesize that the rotational mobility of the dimer may promote transport of polar molecules across the membrane by acting like a hole-drilling device. 

**Figure 14:**
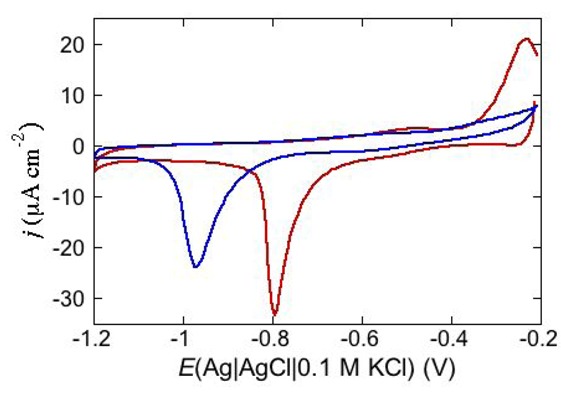
Cycle voltammogram of a DPTL/DOPS tBLM in a pH 7 buffer solution of 0.1 M KCl and 4 μg mL^−1^ DCD at a scan rate of 50 mV/s before (**blue curve**) and after addition of 4 × 10^−5^ M ZnSO_4_ (**red curve**).

Cyclic voltammetry is also one of the most suitable electrochemical techniques to test membrane permeabilization by peptides towards both electroinactive and electroactive ions. Under certain circumstances, permeabilization of a tBLM towards electroinactive ions such as K^+^ or Cl^−^, which are present in biological fluids, may provide a clue to the conformation assumed by a peptide in biological membranes. This is the case with dermcidin (DCD), an antimicrobial peptide present in human sweat [38]. Its elongated helix, about 8 nm long, is twice as long as the thickness of the hydrocarbon tail region of a typical membrane. DCD has a positively charged C-terminal section and a negatively charged N-terminal section, with an overall charge of −2 at pH 7. In view of its length, different conformations in a membrane have been proposed. Paulmann *et al.* [45] postulated a channel structure in which the N-terminal region of DCD folds back into its C-terminal region, forming a transmembrane hairpin, thanks to the flexibility of the DCD *α*-helical structure. Alternatively, the channel was envisaged as a bundle of N-terminal DCD sections embedded in the membrane, with the corresponding C-terminal sections floating on the membrane surface [45]. Conversely, Song *et al.* [46] assumed that the membrane is spanned by a hexameric channel exhibiting its X-ray crystal structure, tilted enough to maintain the channel completely within the membrane. At pH 7, where DCD bears two negative charges, the peptide has no effect at a neutral DPTL/DOPC tBLM [38]. Conversely, at a negatively charged DPTL/DOPS tBLM, where DOPS stands for dioleoylphosphatidylserine, it yields a cyclic voltammogram with a negative peak at about −0.92 V, which is shifted to about −0.75 V in the presence of Zn^2+^, as shown in Figure 14.

It is, therefore, reasonable to hypothesize that this permeabilizing effect is due to the negative charge of the DOPS distal monolayer, possibly in combination with that of DCD. Any DCD conformation completely embedded in the lipid bilayer, such as the extended and tilted conformation proposed by Song *et al.* [46] or the hairpin conformation hypothesized by Paulmann *et al.* [45], is expected to be disfavored by electrostatic repulsion at the DPTL/DOPS tBLM, more than at the DPTL/DOPC tBLM. The conformation in which the positive N terminus penetrates the DPTL/DOPS tBLM at negative transmembrane potentials, while the negative C terminus remains floating on the membrane surface [45], is also expected to be hindered by electrostatic repulsion between the negative C terminus and the DOPS polar heads. However, in this case, Zn^2+^ ion can form a strong zinc bridge between the histidine (His^38^) residue of the C terminus and the carboxyl groups of DOPS. Among other things, at pH 7 His^38^ is expected to be deprotonated, having a pK_a_ of 6.1. Incidentally, this cationic bridge is the basis of the Ni-NTA-His tag technology for protein purification [47]. This procedure consists in fusing a hexahistidine tag (His tag) to the protein and in binding it to a nitrilotriacetic acid group (NTA) via a Ni [48], Zn [49] or Cu ion [50], which is complexed by the three carboxyl groups of NTA and by two histidines. A long peptide chain with a sole histidine residue in a strategic position is a leitmotif of many antimicrobial peptides such as distinctin, magainin and nisin, and it may possibly favor a conformation similar to that proposed for DCD.

The electroactive ions that are more commonly adopted for permeabilization tests at mercury-supported tBLMs are Tl^+^ and Cd^2+^, which exemplify the behavior of monovalent and divalent cations. Both ions are electroreduced reversibly with amalgam formation on uncovered mercury. In aqueous 0.1 M KCl, the Tl^+^/Tl(Hg) and Cd^2+^/Cd(Hg) couples have a formal potential of −0.51 V [12,17] and −0.65 V [17,51], respectively. In the absence of peptides, tBLMs are impermeable to the above cations, as expected. Hence, a current flow following the incorporation of a peptide provides clear evidence of its permeabilizing effect. The current provided by Tl^+^ and Cd^2+^ ions is higher than that due to the movement of electroinactive ions, such as K^+^ and Ca^2+^, across the lipid bilayer moiety of the tBLM. In fact, the current due to these electroactive ions also includes the additional contribution from amalgam formation and is characterized by a negative and a positive peak, with a midpoint between the two peak potentials close to the formal potential of the metal ion/metal amalgam couple.

**Figure 15:**
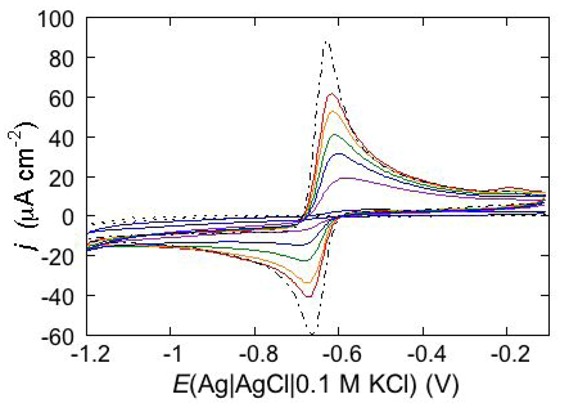
Cyclic voltammograms of 0.20 mM CdSO_4_ in a pH 7 aqueous solution of 0.1 M KCl and of 0.6 μM AmB mixed with Chol in a (1:3) molar ratio, as recorded at a DPTL/DOPC tBLM during continuous potential cycling at a scan rate of 50 mV s^−1^. The curves were obtained upon adding CdSO_4_ before the AmB/Chol mixture. Increasing peak currents correspond to an increasing number of potential cycles. The flat dotted curve was obtained in the absence of the AmB/Chol mixture; the dash-point curve was obtained at a newly formed uncoated mercury drop immersed in a solution of 0.1 M KCl and 0.20 mM CdSO_4_ containing the AmB/Chol mixture.

An interesting example consists in the permeabilization of a tBLM towards Cd^2+^ ion by amphotericin B (AmB), a very powerful antifungal agent that, thanks to its higher efficiency in fungal membranes than in mammalian ones, is used in conventional antifungal preparations. The notable permeabilization of a DPTL/DOPC tBLM to Cd^2+^, with amalgam formation, by 0.6 μM AmB complexed with sterols in a 1:3 molar ratio, as compared with the absence of any effect in the absence of sterols, suggests a direct participation of sterols in ion channel formation [14]. In particular, different results are obtained depending on whether Cd^2+^ is added to a pH 7 buffer solution of 0.1 M KCl before or after the AmB-sterol mixture. When Cd^2+^ is added before the AmB-sterol mixture, continuous potential cycling in the solution of the two species causes a gradual increase in Cd^2+^ electroreduction and Cd(Hg) oxidation peaks (see the solid curves in Figure 15) until ultimately, after several cycles, a stable quasi-reversible cyclic voltammogram is attained. This is similar to the cyclic voltammogram recorded at a newly formed uncoated mercury drop immersed in the same aqueous solution containing the AmB-sterol mixture (dash-point curve in Figure 15), albeit somewhat smaller. Conversely, when the same aqueous solution is obtained by adding the AmB-sterol mixture before Cd^2+^, the reduction current vanishes after a few potential cycles. If another identical aliquot of the AmB-sterol mixture is subsequently added to this solution, further potential cycles determine the same behavior as observed when Cd^2+^ is added before the AmB-sterol mixture, ultimately yielding a stable quasi-reversible cyclic voltammogram. In carrying out the above measurements an important difference is observed, depending on whether cholesterol (Chol) or ergosterol (Erg) are employed in the AmB-sterol mixture. Thus, if after attaining the quasi-reversible cyclic voltammogram by continuous potential cycling between −0.10 and −1.20 V, the potential is repeatedly scanned between −0.20 and −0.80 V, which corresponds to the range of physiological transmembrane potentials, the cyclic voltammogram in the presence of Chol decreases rapidly in height and ultimately vanishes. Conversely, the cyclic voltammogram in the presence of Erg remains stable even in the latter physiological potential range. 

To verify whether the importance of adding Cd^2+^ to the solution before the AmB-sterol mixture, in order to permeabilize the tBLM, is due to Cd^2+^ electroactivity or just to its being a divalent inorganic cation, the above experiments were repeated in a pH 7 aqueous solution of 0.1 M KCl and 2 mM Ca^2+^. In particular, the AmB-sterol mixture was added to this solution before Cd^2+^, stirring the solution after each addition and cycling the potential continuously between −0.10 and −1.20 V. In this case, potential cycling causes a gradual increase in Cd^2+^ reduction and Cd(Hg) reoxidation, until a stable quasi-reversible cyclic voltammogram is attained. The above results can be summarized by stating that ion channel formation in the presence of sterols requires the presence in solution of a freshly added AmB-sterol mixture and of Cd^2+^ or Ca^2+^ ions. It is well known that AmB, once added to a solution bathing a BLM, tends to be adsorbed flat on the surface of the membrane [52,53]. On the other hand, cadmium or calcium ions added to the solution are expected to interact strongly with the phosphate groups of the polar heads of the tBLM.

Evidently, these two different types of interactions prevent an effective direct interaction between AmB-sterol complexes and the divalent cations on the membrane surface. We may tentatively hypothesize that a direct interaction between these two different species in the bulk solution may give rise to a more or less transient complex capable of addressing the AmB-sterol complexes normally to the membrane plane, so as to favor their incorporation in the membrane and subsequent aggregation with ion channel formation. 

**Figure 16:**
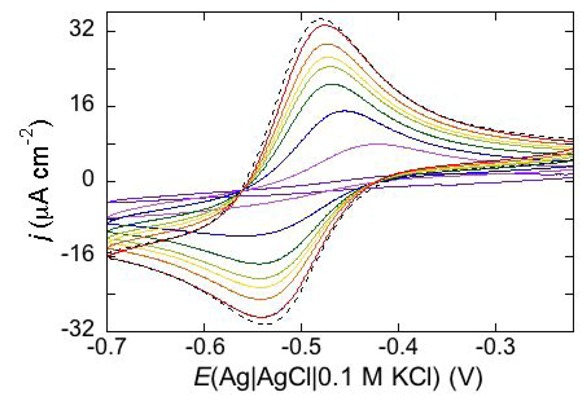
Cyclic voltammograms of 1.4 × 10^−4^ M Tl^+^ in aqueous 0.1 M KCl, recorded on a mercury supported DOPC SAM at different times *t* measured from the addition of 0.2 μM D1. In the order of increasing current, *t* equals 0, 8, 18, 28, 38, 48, 59, 75 min. The dashed curve is the Tl^+^ cyclic voltammogram recorded on bare mercury. Scan rate: 50 mV s^−1^.

Permeabilization tests using the electroactive cations Tl^+^ and Cd^2+^ are also conveniently performed at mercury-supported phospholipid SAMs, since mercury itself provides the incorporated peptides with a large reservoir for the amalgam-forming metal ions. An example is provided by the effect of distinctin (D1), an antibiotic cationic peptide, on DOPC SAM permeabilization to Tl^+^ and Cd^2+^ ions [17]. Figure 16 shows a series of cyclic voltammograms recorded at regular time intervals on a mercury-supported DOPC SAM immersed in an aqueous solution of 0.1 M KCl and 1.4 × 10^−4^ M Tl^+^. To this end, 4 nmoles of D1 were added to the aqueous solution with a microsiringe whose needle was placed at about 1 cm from the mercury drop. This addition induces the appearance of a Tl^+^ cyclic voltammetry curve that is initially low and quasi-reversible, with a separation (∆*E*_p_) between the oxidation and reduction peaks of about 140 mV. With increasing time, the height of the peaks increases and their separation attains a value of 63 mV, close to the value predicted for reversible behavior (59 mV). At long times, D1 tends to make the DOPC monolayer perfectly permeable to Tl^+^ ions.

**Figure 17:**
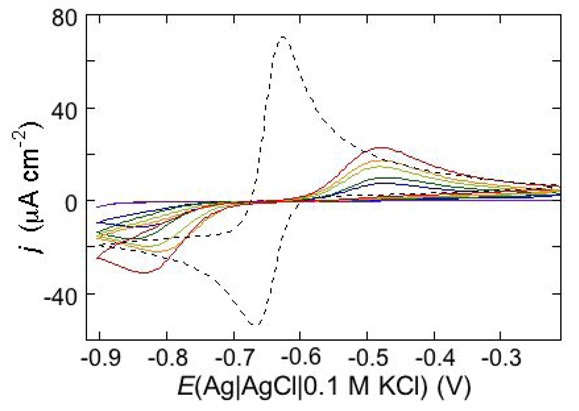
Cyclic voltammograms of 1.5 × 10^−4^ M Cd^2+^ in aqueous 0.1 M KCl, recorded on a mercury-supported DOPC SAM at different times *t* measured from the addition of 0.2 μM D1. In the order of increasing current, *t* equals 0, 6, 14, 23, 33, 43 min. The dashed curve is the Cd^2+^ cyclic voltammogram recorded on bare mercury. Scan rate: 50 mV s^−1^.

If, immediately after the addition of D1 to the Tl^+^ solution in aqueous 0.1 M KCl, the solution is stirred for a few seconds, the cyclic voltammogram recorded at the DOPC SAM becomes practically equal to that recorded on bare mercury in the same solution. This indicates that the progressive increase in the height of the cyclic voltammetry peaks shown in Figure 16 is mainly due to slow D1 diffusion toward the electrode. A rapid addition of D1 to an aqueous solution of 0.1 M KCl + 1.5 × 10^−4^ M CdSO_4_ causes the appearance of a cyclic voltammetry curve of Cd^2+^, which increases in time with an increase in the amount of D1 that reaches the electrode surface, as shown in Figure 17. As distinct from the cyclic voltammogram of Tl^+^, that of Cd^2+^ is totally irreversible, with a ∆*E*_p_ value of about 360 mV at a scan rate of 50 mV s^−1^. Moreover, the peak current attains a maximum limiting value that is decidedly less than that on bare mercury. It should be noted that Cd^2+^ electroreduction is not complete over the potential range of stability (−0.90 V < *E* < −0.20 V) of the Hg-supported DOPC monolayer. This is also true for the reversible cyclic voltammogram of Cd^2+^ on bare mercury, if the same potential range is adopted (see the dotted curve in Figure 17). An inorganic monovalent anion that is electroactive on bare Hg over the above potential range is iodate ion. Thus, it starts to be electroreduced to iodide ion at about −0.35 V in a pH 5.5 phosphate buffer containing 0.1 M KCl. Its electroreduction is completely blocked by a DOPC monolayer. In this case, the presence of D1 has no effect [17]. The above results point to a clear-cut selectivity of the pores created by D1 in lipid monolayers toward inorganic cations. The easier penetration of inorganic monovalent cations with respect to divalent ones is to be expected, in view of the higher potential energy barrier opposed by the hydrocarbon tails of the lipid monolayer to the penetration of ions of higher charge.

## 5. AC Voltammetry

AC voltammetry consists in applying an AC voltage of small amplitude and given frequency to the electrochemical cell, and in recording the quadrature component of the current that flows through the cell with the same frequency, as a consequence of this perturbation. To record the current as a function of the applied potential *E*, the AC voltage is superimposed to a bias voltage that is varied linearly in time. The current is converted into the differential capacitance *C* by calibrating the instrument with a high precision capacitor in place of the electrochemical cell. As long as the adsorbed molecules do not undergo reorientation and no ion movement across the lipid film takes place, the AC voltammogram is flat. Conversely, if the adsorbed molecules undergo a reorientation that causes a change in the component of their dipole moment normal to the lipid film, the potential difference so generated must be compensated for by a flow of electrons along the external electric circuit, to maintain the potential difference across the whole electrified interface constant. This causes a more or less intense pseudo-capacitance peak in the AC voltammogram. The same considerations apply to an ion movement across the lipid film.

AC voltammetry is conveniently employed for investigating the interaction of peptides with the hydrocarbon tail region and, most importantly, with the polar heads of mercury-supported phospholipid SAMs. A self-assembled lipid monolayer can be regarded as the first barrier that a peptide meets in its attempt to penetrate a membrane. Nonetheless, the interaction of the peptide with the hydrocarbon tail region of a SAM is generally different from that with a lipid bilayer, especially if its length is appreciably higher than the SAM thickness, as is often the case. In fact, the peptide can only intercalate partially between the hydrocarbon tails, without being capable of assuming the same conformation as in a biomembrane. Conversely, its interaction with the polar heads of a SAM can reproduce realistically that with a biomembrane.

**Figure 18:**
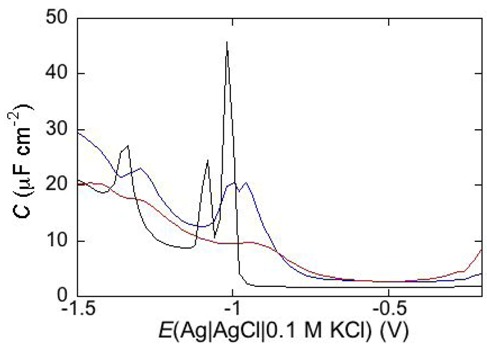
AC voltammograms at 75 Hz of a mercury-supported DOPC SAM in aqueous 0.1 M KCl (black curve), and in aqueous solution of 0.1 M KCl and 2 μg mL^−1^ DCD either unbuffered (red curve) or buffered at pH 7 (blue curve).

A mercury-supported DOPC monolayer in a 0.1 M KCl aqueous solution yields the AC voltammetry curve of the differential capacitance *C* against the applied potential *E* shown by the black curve in Figure 18 [10,14,38]. This phospholipid forms well defined and tightly packed monolayers on mercury, excluding nonspecific ion leakage through monolayer defects in the absence of interacting molecules. Over the potential region of minimum capacitance, which ranges from −0.20 to −0.80 V, the DOPC monolayer is impermeable to inorganic ions, whereas it becomes permeable outside this region. The *C* value over this region amounts to 1.8 μF cm^−2^, namely twice as high the value for a solvent-free BLM. At positive potentials the region of minimum capacitance is delimited by a capacitance increase that precedes mercury oxidation; at negative potentials it is delimited by a sharp pseudocapacitance peak that lies at about −1.02 V, followed by two further peaks at about −1.08 V and −1.35 V, as shown by the black curve in Figure 18. The first two peaks are ascribed to a cooperative reorientation of the lipid molecules, whereas the third one is due to their partial desorption [54]. The first peak results from surface defects that allow a practically uninhibited access of inorganic ions to the mercury surface, while the second peak results from nucleation and growth of the defects formed during the first peak, causing their coalescence. As a rule, species capable of penetrating the hydrocarbon tail region of the phospholipid monolayer increase its capacitance over the potential range of the flat capacitance minimum with respect to its value in the absence of foreign species, if their polarizability is appreciably higher than that of the lipid molecules. Conversely, they affect the monolayer capacitance only slightly if they have a low polarizability, or they may even decrease it if they contribute to thickening or stiffening the monolayer [55]. In both cases, if their concentration in the lipid monolayer is sufficiently high, their intercalation between the lipid molecules prevents the latter molecules from undergoing a sufficiently sharp cooperative reorientation, thus broadening and depressing the pseudo-capacitance peaks exhibited by the black curve in Figure 18. Molecules adsorbed on top of the lipid monolayer, but unable to penetrate it, alter and depress the pseudo-capacitance peaks by interacting with the polar heads, but exert a negligible effect on the capacitance over the (−0.2 V > *E*> −0.8V) potential range [56].

The AC voltammogram of a DOPC monolayer in aqueous 0.1 M KCl is unaltered in passing from an unbuffered solution to a pH 7 buffer solution. Addition of 2 μg/mL dermcidin (DCD) to a pH 7 buffer solution of 0.1 M KCl depresses the pseudo-capacitance peaks of the AC voltammogram and increases the capacitance along the flat region of minimum capacitance, as shown by the blue curve in Figure 18 [38]. This effect is enhanced in an unbuffered solution of 0.1 M KCl, where the pseudo-capacitance peaks are practically suppressed (see the red curve in Figure 18). The depression of the pseudo-capacitance peaks of the DOPC SAM by DCD in the pH 7 buffer solution of 0.1 M KCl and their complete suppression in unbuffered solution denotes a strong interaction between DCD and the polar heads of these phospholipids. This can possibly be explained by the presence of three pairs of contiguous oppositely charged residues in the DCD peptide chain, namely Glu^5^-Lys^6^, Lys^23^-Asp^24^ and Lys^41^-Asp^42^. We can, therefore, envisage a strong dual electrostatic interaction of a negative glutamate (Glu) or aspartate (Asp) residue with the trimethylammonium group of a DOPC polar head and of the contiguous positive lysine (Lys) residue with the negative phosphate group of the same polar head. The complete suppression of the DOPC pseudo-capacitance peaks by DCD in unbuffered solution denotes a stronger interaction of the peptide in this solution than in a pH 7 buffer solution. It is possible that the phosphate ions of the pH 7 buffer interact with the Lys residues of DCD, thus competing with the phosphate groups of the DOPC polar heads.

**Figure 19:**
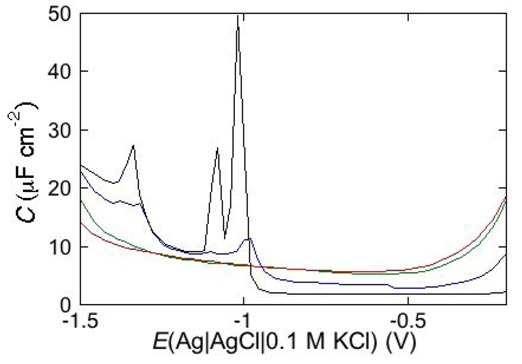
AC voltammograms at 75 Hz of a mercury-supported DOPC SAM in a pH 7 buffer solution of 0.1 M KCl in the absence of AmB (**black curve**), and in the presence of 0.4 (**blue curve**), 0.8 (**green curve**) and 4 μM AmB (**red curve**).

The effect of amphotericin B (AmB) on a DOPC SAM is particularly drastic, up to becoming destructive [14]. Figure 19 shows a number of stabilized *C vs. E* curves at a mercury-supported DOPC SAM in 0.1 M KCl in the presence of different AmB concentrations, together with the curve in the absence of AmB. An AmB concentration of 0.4 μM is sufficient to double the capacitance along the flat minimum and to notably decrease the first two peaks. An AmB concentration of 0.8 μM eliminates all peaks completely, while further increasing the capacitance minimum. In practice, the *C vs. E* curve for 4 μM AmB coincides with that obtained by keeping a newly formed uncoated mercury drop in contact with the same AmB solution, within the limits of experimental error. In fact, AmB is strongly adsorbed on bare mercury in 0.1 M KCl, decreasing its capacitance to an appreciable extent. However, a complete stripping of the DOPC SAM by a 4 μM AmB solution is disproved by the corresponding electrochemical impedance spectrum, which has a sensitivity high enough to distinguish a DOPC monolayer only partly disrupted by a AmB solution from a AmB film adsorbed on bare mercury from the same solution. In the DOPC SAM, initial pores may expand due to the AmB present in the solution, possibly leading to AmB/lipid aggregates, which cause the electrode to be depleted of lipid to an appreciable extent.

## 6. Conclusions

The use of electrochemical techniques such as cyclic voltammetry, EIS and charge transient recordings for the investigation of biological systems is becoming increasingly popular, just as the application of the concepts of electrochemical kinetics and of the structure of electrified interfaces to the interpretation of the electrochemical response. Several efforts are presently made to realize biomimetic membranes consisting of a lipid bilayer anchored to a metal electrode through a hydrophilic spacer (tethered bilayer lipid membranes: tBLMs) and satisfying those requirements of ruggedness, fluidity and high electrical resistance that are necessary for the incorporation of integral proteins in a functionally active state. A unique feature of these biomimetic membranes is the achievement of the maximum possible vicinity of a functionally active membrane peptide or protein to an electrode surface (the electrical transducer). Mercury is a particularly advantageous support for tBLMs, since it imparts to the lipid bilayer a fluidity and lipid lateral mobility not shared by solid supports such as gold and silver. Moreover, the hydrophobic surface of liquid mercury allows a lipid monolayer to readily self-assemble on top of it, with the hydrocarbon tails turned toward mercury and the polar heads turned toward the bathing solution; this allows the interactions of peptides with lipid polar heads to be directly monitored by AC voltammetry.

Mercury-supported biomimetic membranes allow fundamental studies on the function of membrane peptides and proteins, opening the way to the elucidation of structure-function relationships in ligand-receptor and protein-protein interactions. Moreover, being highly insulating and free from pinholes and other defects that might provide preferential pathways for electron and ionic transfer across the lipid bilayer, they may easily characterize ion channel activity. By incorporating therapeutically or diagnostically important natural peptides, these tBLMs have the potential to realize sensors targeting biological analytes. The fabrication of a microelectrode consisting of a mercury cap electrodeposited on a platinum microelectrode about 20 μm in diameter, with a DPTL/lipid bilayer tethered on top of it [57], may open the way to the realization of a microarray platform for highly parallel screening of a large set of drugs and diagnostic targets on channel-forming peptides and proteins. By addressing the single micro-tBLMs of a microarray individually by EIS or patch clamping, it should be possible to monitor in parallel the response of a given channel protein to several drugs. In this connection, Nelson and coworkers have fabricated [58] and patented [59] a wafer-based Pt/Hg microarray coated with a phospholipid monolayer, for the screening of drugs capable of interacting with such a monolayer. Thus, the direct, predominantly electrochemical determination of the function of ion channels at mercury-supported tBLMs and lipid SAMs reconstituted from purified components addresses a strongly felt need for the development of new drug candidates or diagnostic test systems.
